# Chondroblastoma Located in the Anterior Skull Base: A Case Report and Comprehensive Literature Review

**DOI:** 10.1155/crot/9368865

**Published:** 2025-08-07

**Authors:** Mohsen Fazli, Mahdi Khajavi, Sara Mohammadi, Amir Zaker, Mohammad Jalili, Farzin Davoodi, Narges Bazgir, Nader Akbari

**Affiliations:** ^1^Hearing Disorders Research Center, Loghman Hakim Hospital, Shahid Beheshti University of Medical Sciences, Tehran, Iran; ^2^Tehran University of Medical Sciences, Tehran, Iran

## Abstract

**Background:** Chondroblastoma is a rare and benign bone tumor originating from immature chondroblasts. Chondroblastoma typically affects long bones; however, it can also occur in the skull, especially the temporal bone. The anterior skull base is a rare location for this tumor, with only two reported cases.

**Case Presentation:** A 26-year-old woman presented with epiphora in her right eye and progressive proptosis on the same side. She had a previous biopsy that confirmed the presence of a giant cell tumor of the bone and had undergone an unsuccessful endoscopic surgery. A comprehensive endoscopic procedure subsequently revealed a cartilage-producing neoplasm consistent with chondroblastoma.

**Conclusion:** We presented a successful case of surgical resection of a chondroblastoma in the anterior skull base. Additionally, we reviewed the existing literature and previously documented cases.

## 1. Introduction

Chondroblastoma was first described in 1931 by Codman. He reported nine cases of a growth that he called “a giant cell chondromatous tumor of the epiphysis” [1]. Chondroblastoma is a rare type of benign bone tumor that usually occurs at the end of long bones and is aggressive. It originates from immature chondroblasts [2]. This disease typically affects long bones, especially the femur and humerus, and rarely affects flat bones, such as the cranium [3]. Chondroblastoma normally occurs in the temporal bone of the skull [4, 5]. However, other parts of the skull are also affected by chondroblastoma. The anterior skull base is one of the rare places of chondroblastoma in the skull. To our knowledge, only two cases of this lesion in the anterior skull base have been reported [4, 6]. In this study, we report a case of chondroblastoma in the anterior skull, and we reviewed the literature.

## 2. Case Presentation

**Figure 1:**
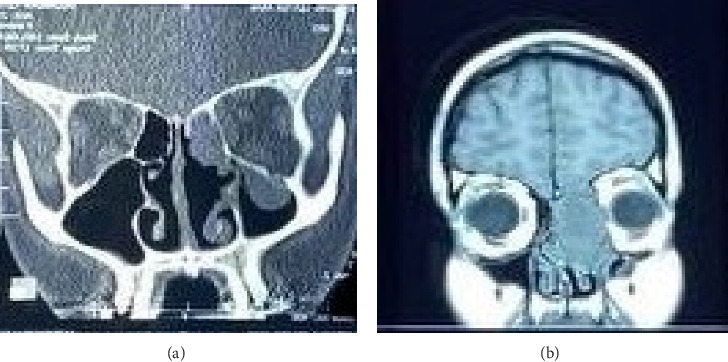
The CT (a) and T1-weighted MRI (b) of the patient before surgery.

**Figure 2:**
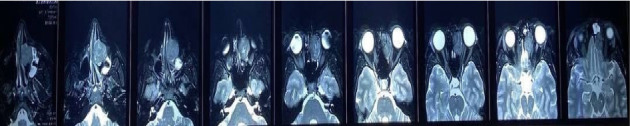
The T2-weighted MRI of presented case.

A 26-year-old woman with no significant medical history has experienced epiphora in her right eye for the past six months, accompanied by gradual protrusion of the right eye (proptosis) for the past year. A comprehensive physical examination, with detailed ophthalmologic and neurological assessments, revealed no abnormalities in the patient's overall health. However, the examination did identify a notable right-sided proptosis characterized by the abnormal protrusion of the right eye. On the paranasal computed tomography (CT) scan, a soft tissue mass caused erosion of the medial wall of the right orbit and cribriform plate and had extended intracranially and right orbit. She had a biopsy and histopathological primary diagnosis of a giant cell tumor of the bone. A mass measuring 48 × 30 × 24 mm was found on magnetic resonance imaging (MRI), heterogeneously enhancing in the right ethmoid air cells, with extensions into the intracranial space, the right orbital region, and mild left paranasal involvement. Concentrated retained secretions of the right frontal and maxillary sinus were also seen. She had a previous unsuccessful surgery in a private center with a remarkable remaining tumor intranasal and intracranially. Figures 1 and 2 show the MRI of patients.

In nasoendoscopy, prominent, lobulated mass obstructing the right nasal cavity and ethmoid sinus was evident, and eventually displaced turbinates. The lesion appeared reddish with mucosal erythema and edema. There was no sign of cystic or hemorrhagic area.

The patient underwent a second comprehensive endoscopic surgery with a guidance system (IGS) under hypotensive general anesthesia. The procedure involved a thorough inspection of the nasal cavity, peripheral ostectomy, and locating a solid soft-tissue tumor that contained cystic components with venous blood. Gauze packing was utilized to manage bleeding during the gradual removal of the mass. Imaging guidance helped pinpoint essential anatomical structures, ensuring that the meninges, lamina papyracea, and internal carotid artery were preserved. The tumor was completely excised.

The tumor was resected piece by piece, with each fragment sent to the pathology ward for evaluation until the margin was clear. A neurosurgeon was also present during the surgery. Because the skull base was involved, reconstruction was necessary. The reconstruction was performed using the triple F method (fat, fascia, and flap). Fat and fascia, derived from the fascia lata, were placed intradurally, while a simple mucosal flap was positioned extradurally.

**Figure 3:**
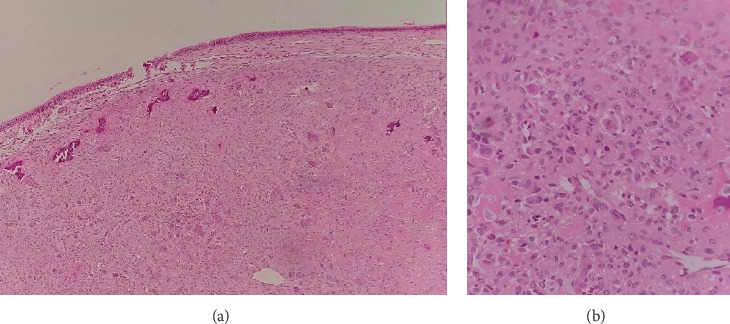
The findings of pathological investigations. The pathology revealed a cartilage-producing tumor with various magnifications. (a) Hematoxylin and eosin ×40 and (b) hematoxylin and eosin ×100.

The histopathological analysis of the resected tumor revealed a cartilage-producing neoplasm consistent with a chondroblastoma diagnosis. Biopsy results of the specimen depicted foci of coarse calcification and areas of pericellular chicken-wire calcification. IHC analysis revealed S100 positivity in the cartilaginous component and DOG1 in some tumor cells.  Figure 3 shows the results of pathological investigations of the patient.

**Figure 4:**
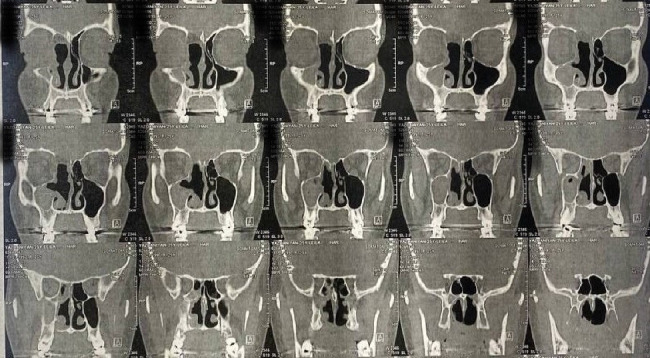
The patient's postoperative CT scan after a year.

The patient experienced no postoperative complications. Epiphora has improved, and there has been no recurrence or residue of the tumor reported in the 1-year follow-up.  Figure 4 shows the postoperative CT scan of the patient.

## 3. Literature Review

Two cases of chondroblastoma in the anterior skull base have been reported previously. The details of these cases are presented in Table 1.

According to Table 1, a common initial symptom observed among patients was eye involvement, particularly a decrease in vision. In all three cases, the masses were located in the ethmoid and sphenoid sinuses. Chondroblastoma appears hypointense on T1-weighted MRI and hyperintense on T2-weighted MRI. All patients underwent surgery, and postoperation, they reported relief from their symptoms without any complications [6, 7].

## 4. Discussion

In the past, chondroblastoma was thought to be completely benign. However, its aggressive nature in the affected area contradicts this belief. As a result, the WHO has adopted a different perspective in its 2013 classification and now considers the disease to be a borderline malignant condition [8].

The exact mechanism of chondroblastoma is not thoroughly understood. Chondroblastoma is a rare type of bone and joint tumor, most commonly arising in the epiphyses of the long bones [8]. This tumor is usually large and is commonly more than 2 cm [9].

The skull is considered an unusual site of occurrence, with most cases arising from the temporal bone [2]. However, chondroblastoma in ethmoid air cells of the skull vault is an extremely rare occurrence [5]. In the literature, there are only two reported cases of chondroblastoma in the anterior cranial base thus far [6, 7].

Chondroblastoma is more common among children [2]. There is a male predominance in chondroblastoma occurrence [5]. However, two of the cases with chondroblastoma in the anterior skull base were female.

It typically impacts people in their first or second decades of life, either in their spine or long bones. As mentioned, cranial chondroblastoma is exceedingly rare, composing less than 2% of cases [5]. Except for the first case, the other two cases were in their third decade of life [6, 7]. Hearing loss, local swelling, otalgia, tinnitus, restricted jaw movement, and pain in the affected area are the most frequent manifestations in patients with cranial chondroblastoma [5]. All discussed cases reported a decrease in visual accuracy and ophthalmic involvement. The other reported manifestations were headache, epistaxis, rhinorrhea, nasal obstruction, proptosis, and epiphora [6, 7].

In radiological evaluations, chondroblastoma is demonstrated as a well-defined, round-to-ovoid lytic lesion with occasional calcified foci. In the skull, chondroblastoma usually occurs in the temporal bone. In temporal bone, lesions can appear solid, osteolytic lesions, and punctuated calcification areas [3, 4, 10–17].

Radiologically, the disease is well-defined and spacious, without any distinct signs. Additionally, this disease often displays a combination of radiolucency and cystic degeneration, along with secondary aneurysmal bone cysts. In higher-grade pathologies, tumors are likely to invade nearby structures such as the dura or brain. The best way to identify the disease is through MRI (5).

MRI lesions appear as low-to-intermediate intensity on T1-weighted sequences and low-to-high intensity on T2-weighted MRI. They also show contrast enhancement, which can be peripheral, homogeneous, or heterogeneous [13, 14, 18, 19]. The same pattern of MRI was observed in all three cases of anterior skull base chondroblastoma.

The diagnostic features of chondroblastoma at the skull base are not yet well-defined because the condition is rare. It likely shares general characteristics with chondroblastoma found in other locations, such as a sclerotic rim and scattered calcification within the lesion. However, these features were not observed in our case [20, 21]. Therefore, differentiating chondrosarcomas from other skull base tumors using solely imaging data would be very challenging.

The main differential diagnosis for chondroblastoma includes giant cell tumor of the bone, chondromyxoid fibroma, chondrosarcoma, and eosinophilic granuloma [11, 22].

Pathology and immunohistochemistry can be valuable in establishing a diagnosis of osteoblastoma. Histologically, chondroblastoma is characterized by chondroblasts with abundant eosinophilic cytoplasm and multinucleated osteoclastic-like giant cells scattered in the cellular areas, as well as a prominent arrangement surrounding the fibrillary eosinophilic matrix. Areas of pericellular calcification in the shape of chicken wire are diagnostic hallmarks. Focally increased mitotic activity may also be seen [21, 23]. The patient exhibited similar pathological findings.

Despite the proposed characteristic imaging and histologic findings, the primary diagnosis of a giant cell tumor in this patient was not straightforward. S100 and DOG1 positivity and chicken-wire calcification are useful findings to differentiate giant cell tumor from chondroblastoma [24–26].

Although very few malignant cases of osteoblastoma have been reported, this tumor has extensive local invasion characteristic features [1]. Therefore, the recommended procedure for chondroblastoma treatment is complete surgical resection with no indication of adjuvant therapy [4, 5].

In conclusion, we reported a rare case of chondroblastoma in the anterior skull base with epiphora and progressive proptosis, which was successfully treated with surgical resection. Besides, we reviewed the previously reported cases and literature.

## Figures and Tables

**Table 1:** The characteristics of reported cases with chondroblastoma in the anterior skull base.

Case demography	Initial manifestation	Location	Computed tomography (CT) scan significant results	Magnetic resonance imaging (MRI) significant results	Pathology	Treatment	Prognosis
Male, 5 years old	Nasal obstruction, rhinorrhea, headache, left-sided epistaxis, decreased vision	Ethmoid and sphenoid sinuses	The soft tissue density with multiple cystic cavities affected the ethmoid and sphenoid sinuses. The lesion has extended into the bilateral orbits and the anterior skull base, causing erosion.	The multiple cystic lesions extended into the medial orbit, affecting the internal wall of the cavernous sinus and occupying the middle and upper meatus of the left nasal cavity. The MRI showed that the mass had low signal intensity on T1-weighted images and high signal intensity on T2-weighted images.	Cyst-like spaces filled with red blood cells, separated by connective tissue septa, and infiltrated with giant cells, spindle-shaped cells, and fibroblasts were observed. Scattered proliferating cartilage cells and chondroblasts were present. The pathological diagnosis was chondroblastoma with a secondary aneurysm and bone cyst.	Surgery	All the symptoms were resolved with no recurrence in follow-ups [6].

Female, 25 years old	Impairment of vision in both eyes and bitemporal hemianopia.	Ethmoid and sphenoid sinuses, tuberculum sellae, sellae, and upper end of clivus	Not mentioned.	The detected mass was hypointense in T1 and hyperintense in T2-weighted MRI. The mass uniformly enhanced with contrast. No para-sellar extension was observed.	Suggestive of chondroblastoma.	Surgery and radiotherapy	A thin layer of residue remained, and the patient was referred to an oncologist for radiotherapy. Afterward, the course was uneventful [7].

Female, 26 years old^∗^	Epiphora from the right eye and progressive right-sided proptosis	The right ethmoid sinus, which caused erosion of the medial wall of the right orbit and cribriform plate, and it extended into the brain cavity and right orbit	Expansive soft tissue mass was seen in the right ethmoid sinus, which caused erosion of the medial wall of the right orbit and cribriform plate, and it extended into the brain cavity and right orbit	A heterogeneous enhancing mass arose in the ethmoid air cells. The mass measured 48 ∗ 30 ∗ 24 mm with intracranial, right orbital, and mild left paranasal extensions	The initial diagnosis was a giant cell tumor of the bone, characterized by focal necrosis and the presence of woven bone and cartilage matrix. Immunohistochemistry (IHC) results indicated negativity for S100. Upon recurrence, the resected tumor was diagnosed as a cartilage-producing neoplasm consistent with chondroblastoma. Biopsy results of the specimen revealed areas of coarse calcification, as well as regions with pericellular chicken-wire calcification.	Surgery	After the second surgery, no recurrence was observed, and the symptoms were relieved.

^∗^The reported case in this study.

## Data Availability

Data are available on request due to privacy/ethical restrictions.
